# Supplementary material for: Molecular elucidation of cement hydration inhibition by silane coupling agents
Source: Nat Commun. 2025 Feb 13;16:1597. doi: 10.1038/s41467-025-56877-9 (PMC11825775; doi:10.1038/s41467-025-56877-9)
Supplement: Supplementary file 1 — Supplementary Information [file 41467_2025_56877_MOESM1_ESM.pdf]

Supplementary Information

Molecular elucidation of cement hydration inhibition by silane coupling agents

Binmeng Chen[a], Meng Wang[a], Hegoi Manzano[b], Yuyang Zhao[a], Yunjian Li[c,*]

[a] Institute of Applied Physics and Materials Engineering, University of Macau, Avenida da Universidade, Taipa, Macao SAR, China

[b] Physics Department, Faculty of Science and Technology, University of Basque Country UPV/EHU, Barrio Sarriena s/n, 48940 Leioa, Spain

[c] Faculty of Innovation Engineering, Macau University of Science and Technology, Avenida Wai Long, Taipa, 999078, Macao SAR, China

* Corresponding author, Yunjian Li. Email: liyunjian@must.edu.mo

Supplementary Note 1: Details of atomic models

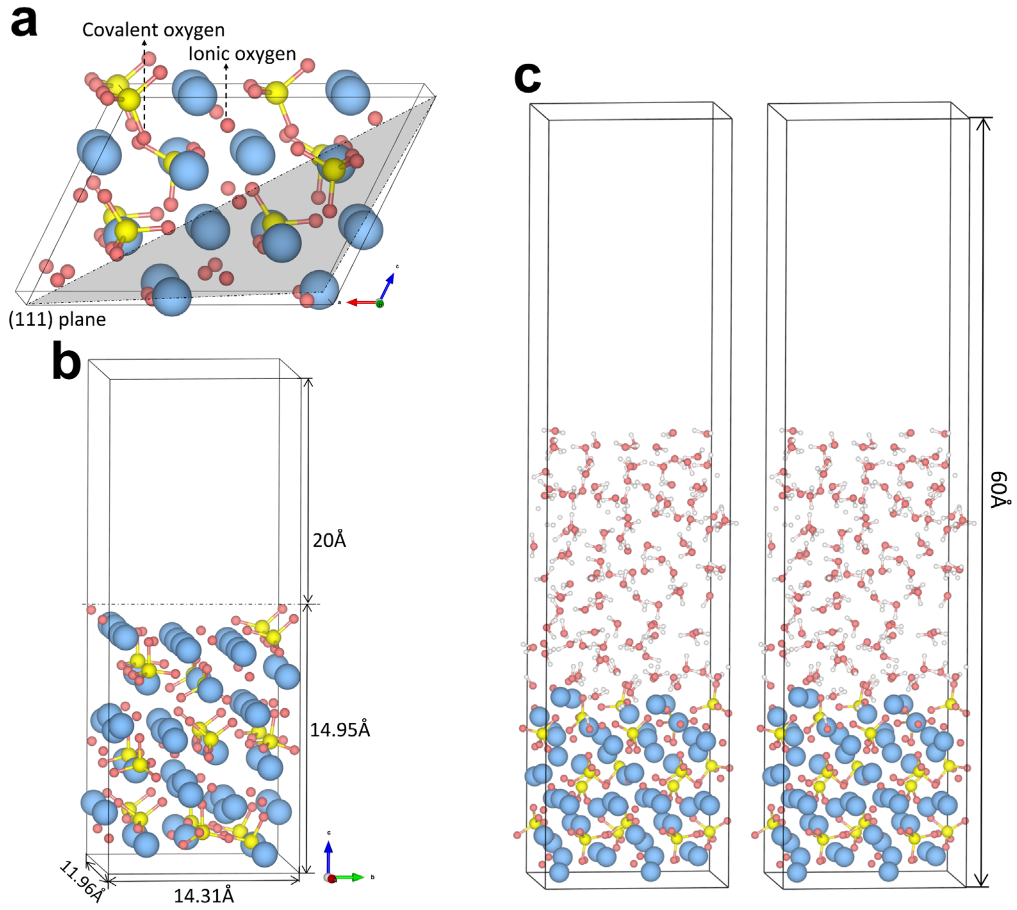

Supplementary Fig. 1 Details of Ca_3SiO_5 atomistic models' construction. **a** M3-Ca_3SiO_5 unit cell structure after geometric optimization. **b** optimized Ca_3SiO_5 slab for adsorption calculation and AIMD simulations. **c** The surface models incorporating both water and an APTES molecule, captured before WT-MetaD simulations.

The unmodified system exhibited a hydroxyl density of 8.0 OH/nm², while the silane-contained system demonstrated a lower density of 4.8 OH^-/nm². In comparison, a ReaxFF study reported a hydroxyl density of 7.0 OH/nm² on the Ca_3SiO_5 surface after the initial hydration stage (at approximately 0.3 ns) [1]. The OH density results indicate that the Ca_3SiO_5 slab has undergone what is referred to as "complete hydrolysis".

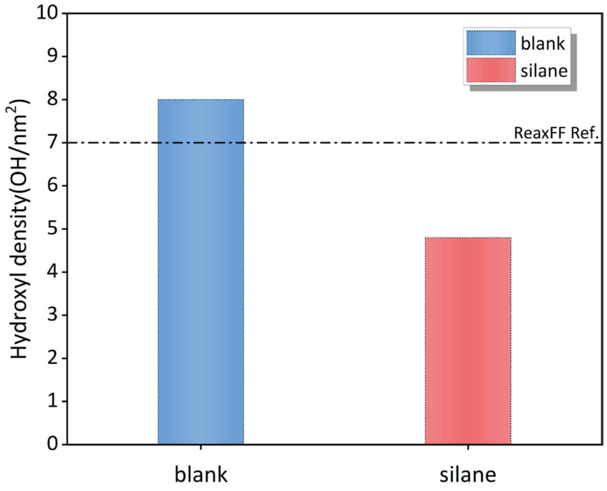

Supplementary Fig. 2 The OH^- density of relaxed Ca_3SiO_5 surfaces.

Supplementary Note 2: Computational equation

The adsorption energy (E_{ads}) was calculated by equation (1) to evaluate the stability of the two modes[2]:

$$E_{ads} = E_{(ad+sub)} - E_{ad} - E_{sub} \qquad (1)$$

Here, E_{ads} denotes the adsorption energy, $E_{(ad+sub)}$ represents the total energy of the entire system after optimization, E_{ad} corresponds to the energy of the APTES molecule, and E_{sub} refers to the energy of the Ca_3SiO_5 slab.

$$g(r) = \frac{\mathrm{d}N}{4\pi r^2 \mathrm{d}r} / \rho \qquad (2)$$

wherein N is the aggregate count of molecules constituting the system under consideration, ρ represents the characteristic density of the said system, and r signifies the spatial separation between two discrete particles therein.

Supplementary Note 3: Convergence tests of WT-MetaD simulations

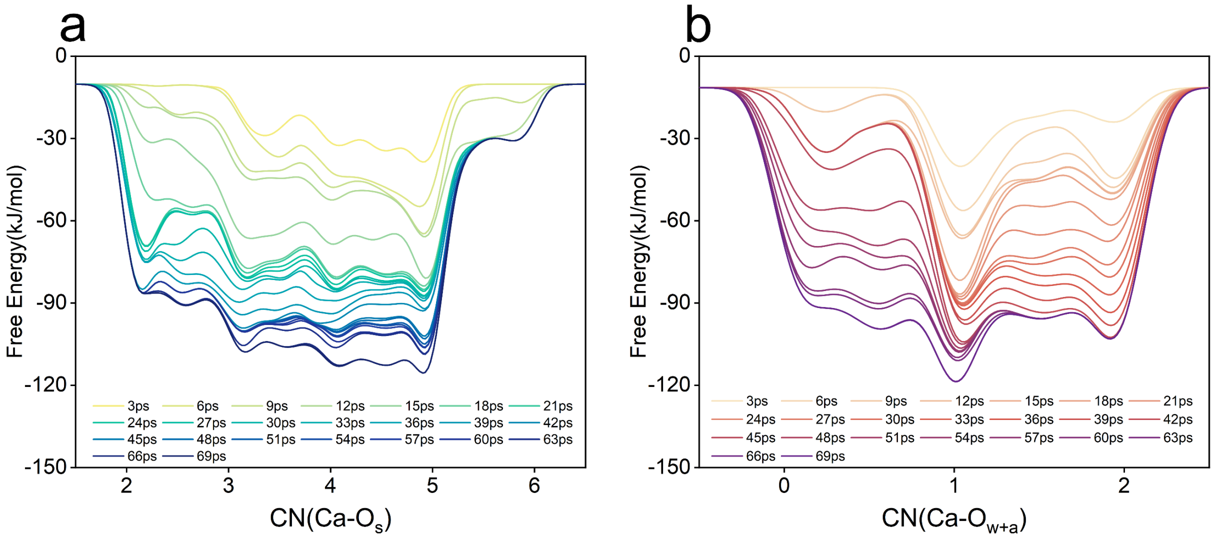

Supplementary Fig. 3 Covergence tests for WT-MTD simulations of silane-containing system. a and **b** display the one-dimensional free energy profiles as a function of CN(Ca-O_s) or CN(Ca-O_{w+a}), sampled at intervals of 3 ps (corresponding to the deposition of 100 Gaussian kernels), throughout the duration of the simulation.

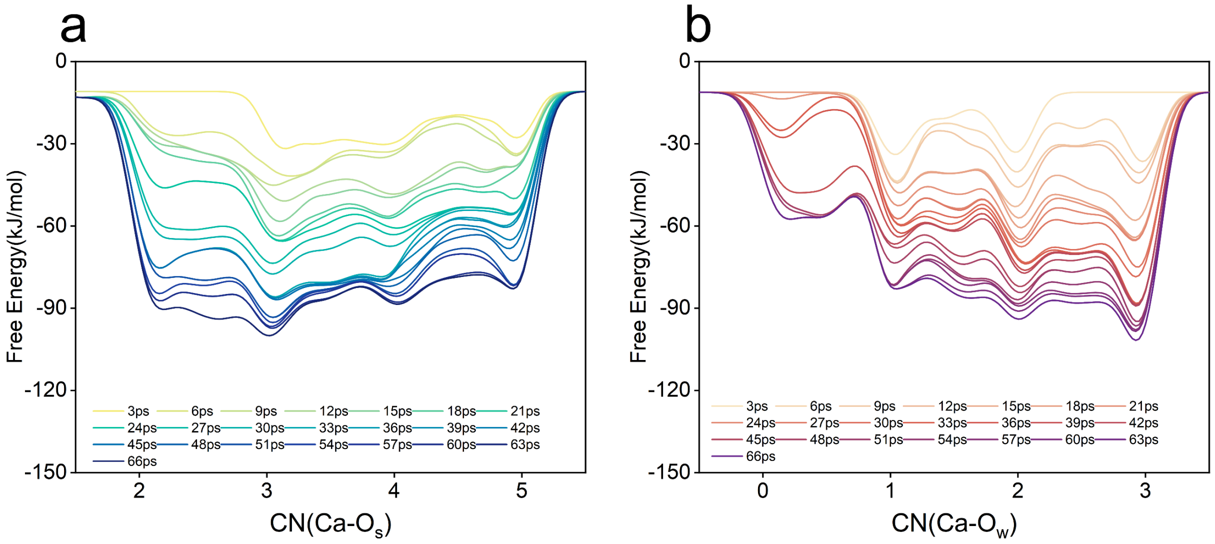

Supplementary Fig. 4 Covergence tests for WT-MTD simulations of the pure water system. a and **b** display the one-dimensional free energy profiles as a function of CN(Ca-O_w) or CN(Ca-O_s), sampled at intervals of 3 ps (corresponding to the deposition of 100 Gaussian kernels), throughout the duration of the simulation.

Supplementary Note 4: Experimental data

Supplementary Table 1 Quantitative data of XPS including coordinates and intensities of diffraction peaks.

Parameters	Binding energy (eV)				Intensity (cps)			
Group	Silane-treatment		Blank		Silane-treatment		Blank	
Orbitals	Ca*2p*	Si*2p*	Ca*2p*	Si*2p*	Ca*2p*	Si*2p*	Ca*2p*	Si*2p*
10 min	347.2	101.7	346.7	101.0	29879.2	2852.26	43981.3	5444.31
30 min	347.0	101.2	346.7	101.1	34605.1	3887.22	37633.9	4723.17
60 min	346.9	101.2	346.7	101.1	35542.3	4008.84	45077.5	5652.65
120 min	346.8	101.2	346.8	101.3	39105.9	4773.82	31430.1	4728.02

Supplementary Table 2 Chemical compositions of C_3S determined by XRF (wt. %)

Series	CaO	SiO_2	Al_2O_3	SO_3	P_2O_5
C_3S	83.2050	16.5655	0.1654	0.0410	0.0231

Supplementary Note 5: Total simulation time of AIMD simulations

Supplementary Table 3 Production time of AIMD simulations

Production details	Time
Surface relaxation after explicit solution introduced	10 ps
Dissolution pathway of Ca under APTES influence	66 ps
Dissolution pathway of Ca in explicit aqueous solution	63 ps
Configuration analysis of final state relaxation from WT-MetaD	10ps for each system

Supplementary reference

1. Huang, J. *et al.* Electronic origin of doping-induced enhancements of reactivity: case study of tricalcium silicate. *J. Phys. Chem. C* **119**, 25991–25999 (2015).
2. Schimka, L. *et al.* Accurate surface and adsorption energies from many-body perturbation theory. *Nat. Mater.* **9**, 741–744 (2010).
